# *Auricularia auricula* Polysaccharide Modulates Rheological, Thermal, and Structural Properties of Wheat Gluten via Selective Regulation of Glutenin and Gliadin

**DOI:** 10.3390/foods15010136

**Published:** 2026-01-02

**Authors:** Haowei Li, Jialu He, Yingxu Liu, Xiaolong Liu, Tingting Liu

**Affiliations:** 1 School of Food Science and Engineering, Jilin Agricultural University, Changchun 130118, China; a1484908375@163.com (H.L.); 13180675546@163.com (J.H.); 118242887096@126.com (Y.L.); 15030377650@163.com (X.L.); 2 Scientific Research Base of Edible Mushroom Processing Technology Integration of Ministry of Agriculture and Rural Affairs, Changchun 130118, China

**Keywords:** *Auricularia auricula* polysaccharide, wheat gluten, glutenin, gliadin, rheological properties, thermal characteristics, structural properties

## Abstract

This study investigated the effects of *Auricularia auricula* Polysaccharide (AAP) concentrations on the rheological and thermal properties of gluten and its subunit components. We used multiple techniques, including dynamic rheology, differential scanning calorimetry (DSC), Fourier-transform infrared spectroscopy (FT-IR), free thiol group analysis, and scanning electron microscopy (SEM). The results revealed that AAP increased the storage (G′) and loss (G″) modulus of gluten, glutenin, and gliadin, promoting compact elastic protein networks. DSC and free thiol group analysis demonstrated that AAP enhanced thermal stability and disulfide bond cross-linking in gluten and glutenin, but reduced thermostability and inhibited disulfide formation in gliadin. Secondary structure analysis showed 31.93% and 17.72% increases in α-helix and β-sheet content, respectively, in glutenin at 8% AAP, thereby enhancing the orderliness of the gluten structure and improving structural rigidity, while reducing gliadin’s structural order. Microscopy confirmed AAP narrowed gluten matrix pores, forming uniform honeycomb structures (though high concentrations caused disruption). In summary, AAP primarily stabilizes gluten conformation by modulating glutenin structure, thereby enhancing rheological and thermal properties.

## 1. Introduction

Gluten is the main protein in wheat dough. It gives dough extensibility, viscoelasticity, and water-holding capacity. Gluten provides structural support and forms the key network determining flour product quality. It accounts for 9–14% of the wheat flour content [1]. Gluten consists mainly of glutenin and gliadin. Glutenin forms a fibrous polymer network through disulfide bonds [2]. Gliadin, by contrast, is composed of monomeric spherical proteins that contribute to its viscosity. When hydrated together, they create a three-dimensional network, acting as the “structural skeleton” of flour products [3]. As demand for high-quality, functional flour products grows, modifying gluten’s structure to improve processing adaptability is a major focus in food science.

Polysaccharides are widely used as gluten modifiers due to their ability to regulate protein-water interactions and intermolecular forces. For instance, carboxymethyl cellulose (CMC) enhances gluten matrices by increasing α-helix/β-sheet content and storage modulus via hydrogen bonding [4], while apple pectin stabilizes gluten by promoting more stable disulfide bond conformations [5]. However, these and other common polysaccharides exhibit inherent limitations.

First, there is a notable trade-off between modification efficiency and bioactivity: highly efficient modifiers, such as CMC, often lack bioactivity, whereas bioactive ones, like inulin or konjac glucomannan (KGM), can disrupt gluten microstructure and show reduced effectiveness [6,7]. Second, our understanding of how polysaccharides interact with specific gluten subunits (glutenin and gliadin) remains limited. This knowledge gap hinders the precise regulation of gluten’s hierarchical structure, from secondary conformation to micro-network formation, making targeted quality improvement challenging.

*Auricularia auricula* polysaccharide (AAP), an anionic hydrocolloid from edible fungi, presents a distinctive profile that may address these dual challenges. Structurally, its backbone of β-(1→3)-D-glucosidic bonds [8] is conducive to strong hydrogen bonding with gluten while minimizing water competition. Functionally, AAP combines bioactivity (e.g., antioxidant, lipid-lowering effects) with apparent modification efficiency [9,10], as low doses (2–8%) can improve dough viscoelasticity and reduce noodle cooking loss [11,12]. However, existing research on AAP-gluten interactions is fragmented. Most studies focus on whole gluten rather than its subunits, leaving it unclear whether AAP selectively modulates glutenin or gliadin. Moreover, the systematic mechanism by which AAP influences disulfide bond rearrangements, secondary structure, and micro-network formation remains unclear.

To address these gaps, this study comprehensively investigates the effects of AAP on gluten and its subunits (glutenin/gliadin) using multi-dimensional techniques (rheology, differential scanning calorimetry (DSC), Fourier-transform infrared spectroscopy (FT-IR), scanning electron microscopy (SEM), and chemical interaction analysis). The specific objectives are (1) to reveal the concentration-dependent regulation of AAP on gluten’s rheological properties and thermal stability, distinguishing its effects on glutenin and gliadin, and (2) to elucidate the molecular interaction mechanisms, including changes in non-covalent/covalent bonds and their correlation with secondary structure and surface hydrophobicity. This work aims to clarify the role of AAP in modulating gluten, revealing its potential for enhancing flour product quality and providing a scientific foundation for its innovative application in the food industry.

## 2. Materials and Methods

### 2.1. Extraction of Gluten, Glutenin, and Gliadin

Following the modified procedure by Liu [6], gluten was extracted from 300 g of raw flour using a 0.4 M NaCl solution. The dough formed after mixing was washed with 0.4 M NaCl solution, then freeze-dried, ground, and sieved (100-mesh). Gluten was dispersed in 65% ethanol and stirred magnetically for 3 h. Subsequent centrifugation separated the gliadin-containing supernatant from the glutenin precipitate, both of which were freeze-dried. Protein purity was determined by Kjeldahl nitrogen analysis, yielding gluten, glutenin, and gliadin contents of 87.17%, 80.39%, and 93.12% (dry basis), respectively.

### 2.2. Preparation of AAP

Crude acidic polysaccharides (AAPs) were obtained from black fungus by following a modified method by Li [11]. Following initial high-pressure water extraction (1.2 MPa, 120 °C, 60 min), the extract was clarified by centrifugation and filtration. Proteins were removed using Sevage reagent (20% *v*/*v*, n-butanol:chloroform 1:4), and subsequent standing and centrifugation further removed protein precipitates. AAP was precipitated from the supernatant with 95% ethanol (6:1 *v*/*v*), collected, concentrated by rotary evaporation, and freeze-dried. The purity of AAP was determined by the phenol-sulfuric acid method. The result was calculated based on the curve Y = 0.12786x + 0.1052 (R^2^ = 0.9972).was 85.72%, with a yield of 5.4%.

**Figure 1:**
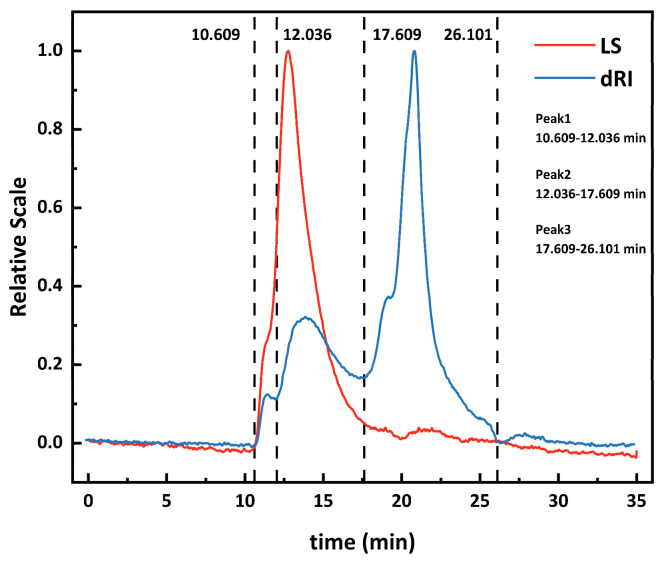
Molecular weight distribution of *Auricularia auricula* polysaccharide (AAP) determined by HPSEC-MALLS-RI.

Polysaccharide molecular weight (Mw) was determined by using the tandem detection method of (HPSEC-MALLS-RI). As shown in Figure 1, the molecular weight distribution of AAP was characterized as follows: weight-average molecular weight (Mw) = 1.457 × 10^6^ g/mol, number-average molecular weight (Mn) = 1.423 × 10^6^ g/mol, and polydispersity index (PDI) = Mw/Mn = 1.024. The molecular weight distribution of AAP was found to consist of three distinct peaks with molecular weights of approximately 1.46×10^6^ g/mol (Peak 1, retention time: 10.609–12.036 min), 6.24 × 10^5^ g/mol (Peak 2, retention time: 12.036–17.609 min), and 5.34 × 10^4^ g/mol (Peak 3, retention time: 17.609–26.101 min).

### 2.3. Sample Preparation

Sample preparation followed the method of [13] with minor adaptations. Protein powders (gluten, glutenin, gliadin; 1 g each) were mixed with 1.5 mL of AAP solutions at varying concentrations (0%, 2%, 4%, 6%, 8%, 10% *w*/*v*) and stirred to achieve homogeneity. After a 40-min room temperature incubation, samples were either subjected to rheological tests or further processed by lyophilization, pulverization, and sieving (100-mesh).

### 2.4. Determination of Rheological Properties

Rheological properties were assessed using an MCR302 rheometer (Anton Paar, Vienna, Austria) equipped with a 50 mm parallel plate sensor (PP-50/P2), following a method adapted from [14]. After a 5-min equilibration, samples underwent a strain sweep (25 °C, 10 Hz; 0.01–100%). Frequency sweeps were then conducted at 25 °C and 0.5% strain (0.01–100 Hz) to record storage modulus (G′), loss modulus (G″), and loss tangent (tanδ).

### 2.5. Determination Thermal Properties

The thermodynamic properties of the experimental operations of the protein were followed by [15]. We exactly weighed 2–3 mg of the freeze-dried protein sample and sealed it in a small aluminum DSC pot. The sample was heated from 20 °C at a rate of 5 °C/min to 80 °C. The denaturation peak temperature (Tp) and enthalpy (ΔH) were analyzed and calculated using the TA system Muse software (version 1.6).

### 2.6. Detection of Chemical Interactions

Non-covalent bond content was determined using a modified method from Qu et al. [16]. Protein samples (200 mg) were incubated with 10 mL of four solutions (PA: 0.05 mol/L NaCl, PB: 0.6 mol/L NaCl, PC: 0.6 mol/L NaCl + 1.5 mol/L urea, PD: 0.6 mol/L NaCl + 8 mol/L urea) in phosphate buffer (0.05 mol/L, pH = 7.0) for 1 h at 25 °C, followed by centrifugation (8000× *g*, 20 min). Soluble protein in the supernatant was quantified using Coomassie brilliant blue with bovine serum albumin as the standard. Ionic, hydrogen bond, and hydrophobic interactions were calculated as the differences in soluble protein between PA/PB, PB/PC, and PC/PD, respectively.

### 2.7. Free Sulfhydryl Group Content

100 mg of Sample powder was combined with 4 mL of reaction buffer (prepared as described) and homogenized thoroughly using a vortex mixer. Subsequently, 40 μL of Ellman’s reagent (4 mg/mL 5, 5′-dithio-bis-2-nitrobenzoic acid) was introduced, and the reaction proceeded for 30 min at 25 °C under light-protected conditions. The mixture was then centrifuged (4000× *g*, 30 min), and the absorbance of the resulting supernatant was read at 412 nm. Quantification was performed using a reduced glutathione standard curve.

### 2.8. Surface Hydrophobicity

Surface hydrophobicity was assessed by the bromophenol blue (BPB) binding assay [17]. A solution of 4 mg/mL sample was incubated with 1.5 mL of 1 mg/mL BPB at room temperature for 1 h. Following centrifugation (12,000 rpm, 10 min), the supernatant was diluted 15-fold and its absorbance measured at 595 nm. A blank control was prepared using deionized water. The bound BPB (BPBbound, µg) was calculated to represent the degree of surface hydrophobicity.

$$BPBbound\;(µg)=300\times \frac{A_{control}-A_{sample}}{A_{control}}$$

In the formula, $A_{control}$ represents the absorbance value of the blank control, while $A_{sample}$ represents the absorbance value of the protein sample.

### 2.9. Fourier Transform Infrared Spectroscopy (FT-IR)

Secondary structure content was determined by FTIR spectroscopy [18]. Samples were prepared by mixing 10 mg of powder with 1.0 g KBr and compressing it into a pellet. FTIR spectra were recorded (400–4000 cm^−1^) and analyzed using Peakfit software (v4.12) to quantify secondary structure elements.

### 2.10. Measurement of Intrinsic Fluorescence Spectra

The methodology of fluorescence spectroscopy was derived from Wouters [19], incorporating minor modifications. The intrinsic fluorescence spectra of the gluten samples were obtained by exciting the protein suspension (200 μL) dissolved in 50 mM acetic acid (1 mg/mL) at 280 nm using an F-7000 fluorescence spectrophotometer (Hitachi, Ltd., Tokyo, Japan), with the emission wavelength ranging from 310 to 410 nm and a slit width of 5 nm.

### 2.11. SEM Observations

The sample was cut into thin slices with a knife and then freeze-dried. After freeze-drying, the sample was subjected to ion sputtering and gold spraying, with the acceleration voltage set at 3.0 kV. Examined at 400× magnification using SEM (Phenom-World BV, Eindhoven, The Netherlands).

### 2.12. Statistical Analysis

Using SPSS (version 27.0; SPSS Inc., Chicago, IL, USA), data were processed and expressed as mean ± SD. Multiple means comparisons employed one-way ANOVA. Differences were deemed significant at *p* < 0.05.

## 3. Result and Discussion

### 3.1. Dynamic Rheological Results

**Figure 2:**
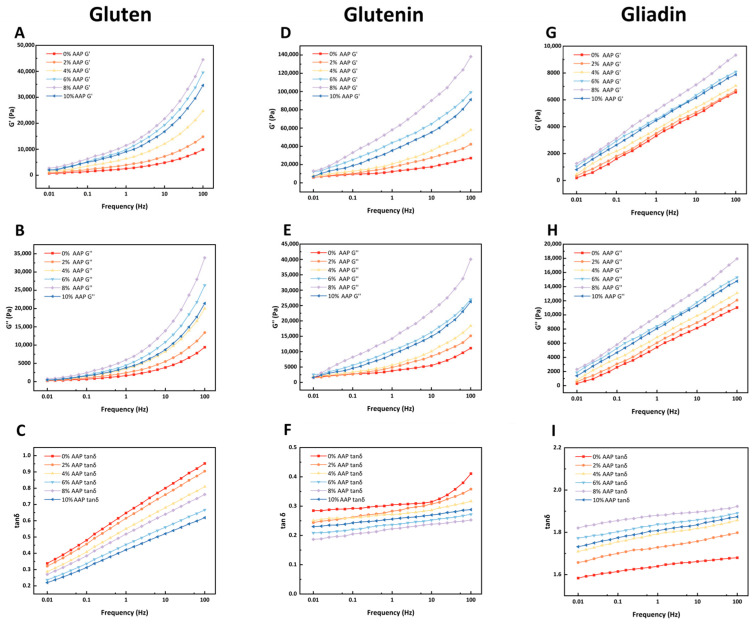
The influence of different amounts of AAP addition on the rheological properties of gluten, glutenin, and gliadin. (**A**,**D**,**G**): Storage modulus (G′) versus frequency. (**B**,**E**,**H**): Loss modulus (G″) versus frequency. (**C**,**F**,**I**): Loss tangent (tanδ) versus frequency.

Figure 2 presents the storage modulus (G′) and loss modulus (G″) of gluten, glutenin, and gliadin as they vary with frequency sweep. The tangent of the loss angle (tanδ = G′’/G′) is also shown. AAP increased both the G′ and G″ values of gluten and glutenin. The tanδ value for gluten was significantly lower than that of the control, indicating improved elasticity. However, at 10% AAP, both viscosity and elasticity decreased. Gliadin exhibited a concentration-dependent response to AAP, with maximum G′ and G″ values observed at 8%. At 10% AAP, these values decreased but remained higher than those of the control. The tanδ value for gliadin also decreased at higher AAP concentrations. Overall, gliadin exhibited lower G′ and G″ values compared to both gluten and glutenin.

The increase in G′ and G″ values of gluten with AAP addition suggests the formation of a more robust gluten matrix. This reinforcement is likely attributed to interactions between AAP and gluten, including hydrogen bonding and van der Waals forces, which reinforce the gluten matrix [4]. In line with the findings of Wang [20], who observed increased G′ and G″ with TP addition.

The G′ and G″ values of glutenin increased with AAP concentration, indicating a more compact network structure. This is likely due to the hydration behavior of AAP, which alters the hydration environment of the system, making the free sulfhydryl (-SH) groups of glutenin molecules more susceptible to oxidation and the formation of disulfide bonds (S-S) [11,21]. Enhanced disulfide bond formation in glutenin is crucial for forming a strong, elastic network. By promoting the conversion of free thiols to disulfide bonds, AAP effectively strengthens these inter-chain connections, leading to a higher G′ and thus a more elastic dough structure. However, at 10% AAP, both viscosity and elasticity decreased. It is inferred that glutenin molecules are encapsulated within this polysaccharide network, thereby inhibiting the oxidative cross-linking of -SH into S-S [22].

The G′ and G″ values of gliadin exhibit a concentration-dose effect with AAP, and reach the maximum at 8%, which may be due to the fact that gliadin contains a large number of glutamine residues; the hydroxyl and carboxyl groups of AAP can form hydrogen bonds with them, significantly enhancing the molecular viscosity [23]. Meanwhile, the progressive increase in AAP concentration enhances the number and dimensions of junction zones between AAP molecules, and this augmentation in cross-linking promotes the self-assembly of the polysaccharides into a cohesive network, consequently leading to a rise in both the G′ and G″ moduli [23].

However, rheological analysis revealed a more pronounced increase in the G″ compared to the G′ for gliadin, which may be related to its structure. Gliadin with a monomer structure mainly forms loose molecular aggregates through non-covalent interactions and lacks a three-dimensional network of intermolecular S-S cross-linking, and imparts viscosity to the dough [3]. Therefore, we speculate that, due to the structural differences in gliadin, it is unable to form a dense three-dimensional network structure through intermolecular S-S and non-covalent interactions, and thus cannot closely combine with AAP. The solid properties and G′ of the gliadin have not been significantly enhanced. When the AAP concentration is 10%, the G′ and G″ values of gliadin are lower than those at 8%, but still higher than those of the control group. The decrease in tanδ value indicates that high concentrations of AAP weaken the viscosity of gliadin and have a destructive effect on the gliadin structure. It is possible that, during the binding process of gliadin-AAP, AAP has strong water retention properties. It competes for water with the gliadin [20], thereby interfering with the formation of gliadin.

In summary, AAP enhances the G′ and G″ values of gluten and its subunit components, with the highest values observed at 8%. This indicates that AAP strengthens the gluten network and stabilizes its structure. However, gliadin’s contribution to the rheological behavior remains limited, as reflected in its lower G′ and G″ values compared to gluten and glutenin.

### 3.2. Thermal Properties

As shown in Table 1, the addition of AAP significantly increased the characteristic temperature (Tp) of gluten and glutenin compared to the control sample. This indicates that AAP improved the thermal denaturation resistance and promoted a more stable and resilient structure of these proteins. This aligns with the rheological findings. In contrast, the Tp of gliadin decreased with increasing AAP concentration, reaching a minimum at 10% AAP (a 7.97% decrease compared to the control). With increasing AAP concentration, the ΔH of gliadin decreased significantly, indicating a reduction in the orderliness of its structure. Conversely, the ΔH of gluten and glutenin initially increased and then decreased with rising AAP concentration.

The observed increase in Tp for gluten and glutenin upon the addition of AAP. This effect is likely attributable to AAP’s high hydrophilicity, which promotes strong anchoring of water molecules via hydrogen bonds. This process may lead to gluten dehydration, subsequently strengthening the interactions between gluten molecules and requiring a higher temperature for denaturation [24]. It is also possible that AAP’s ability to promote the formation of a more ordered and stable protein structure. AAP induces the conversion of β-turns to β-sheets in protein, which is known to enhance the stability of the protein structure, thereby increasing thermal stability [25]. The β-sheet content is proportional to the viscoelasticity and hardness of the dough, as reported by Pessato et al. [25], and the increase in β-sheets observed at 8% AAP directly contributes to the improved thermal stability of glutenin. Nawrocka [5] also observed a similar phenomenon. They found that the addition of MCC, IN, AP, and CP increased the denaturation temperature of gluten. However, it is important to note that a high concentration of AAP might hinder gluten matrix formation and reduce its overall thermal stability. The decrease in the Tp of gliadin with increasing AAP concentration suggests a disruption of its native structure. The AAP may induce the formation of a large number of β-turns in gliadin, altering its ordered structure and reducing its thermal stability.

ΔH not only reflects the degree of protein aggregation but also represents the frozen water content that can be frozen [26]. The elevated ΔH in gluten and glutenin at lower AAP concentrations might suggest that AAP promotes their aggregation and increases structural orderliness. This could be related to conformational changes in polysaccharides within gluten and glutenin, which enhance hydrophobic interactions, driving protein aggregation and influencing peptide chain rearrangement [27]. For instance, konjac glucomannan (KGM) has been shown to enhance gluten aggregation by strengthening protein interactions, thereby delaying thermal denaturation [28].

The initial higher ΔH of gliadin compared to gluten and glutenin is consistent with its known functional characteristics. Due to its high content of polar amino acids, gliadin readily hydrates in dough, contributing to extensibility, while the glutenin network provides water retention [29,30]. The significant decrease in gliadin’s ΔH with AAP addition implies a reduction in its structural orderliness, potentially due to the structural changes induced by AAP.

### 3.3. Chemical Interactions Analysis in AAP-Protein Composite System

**Figure 3:**
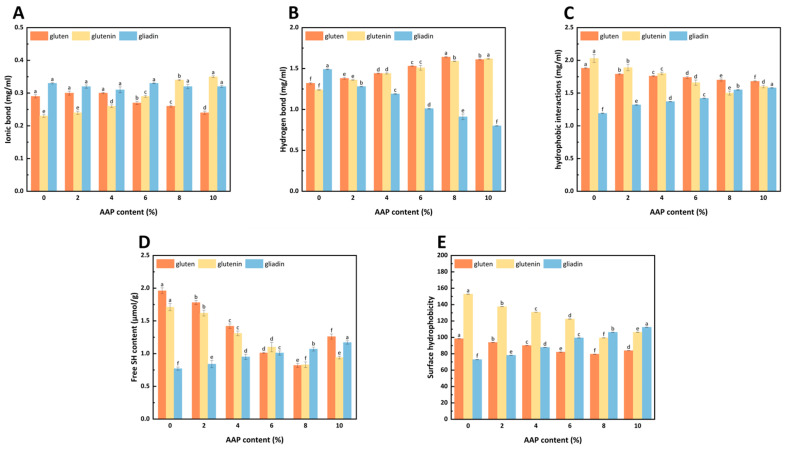
The effects of different AAP addition amounts on the non-covalent interactions of gluten, glutenin, and gliadin. ((**A**–**C**) represent the relationship diagrams of the contents of ionic bonds, hydrogen bonds, and hydrophobic interactions with AAP addition amounts). (**D**,**E**) represent the effects of different AAP addition amounts on the surface hydrophobicity and free thiol content of gluten, glutenin, and gliadin. Different lowercase letters indicate significant differences, *p* < 0.05.

As shown in Figure 3, the ionic bond strength in gluten decreased with increasing AAP concentration. Conversely, in glutenin, the ionic bond strength increased with the addition of AAP. AAP significantly strengthened hydrogen bond binding in gluten and glutenin. However, it weakened the hydrogen bond stability of gliadin. Notably, at very high AAP concentrations, the hydrogen bond binding strength of gluten decreased. AAP enhanced hydrophobic interactions in gliadin while weakening them in gluten and glutenin.

The different effects of AAP on the intermolecular forces between gluten, glutenin, and gliadin molecules are related to their structures. The crucial role of hydrophobic interactions in gluten and glutenin, in contrast to the dominance of hydrogen bonding in gliadin, is likely due to their distinct amino acid compositions [31]. Gluten and glutenin may contain a higher proportion of hydrophobic amino acids, leading to stronger hydrophobic interactions, whereas gliadin’s richness in polar amino acids favors hydrogen bonding.

The weakening of ionic bonds in gluten with AAP addition, while strengthening in glutenin, suggests that AAP’s charged groups interact differently with the exposed charged residues of these two protein fractions. The decrease in ionic bond strength might be AAP’s negative charge (from carboxyl and sulfate groups), which allows it to interact electrostatically with positively charged amino acid residues in gluten, promoting structural changes [32]. Liu [33] found that medium- and high-molecular-weight inulin reduced the ionic bond interaction in gluten, indicating that inulin interfered with the electrostatic interaction with gluten. For glutenin, AAP promotes the formation of a rigid polymer matrix via intermolecular disulfide bonds. This process could force the charged regions of glutenin into closer contact, thereby reducing the influence of polysaccharides on its surface-charged residues and consequently strengthening the ionic bonds [34].

As shown in Figure 3B, AAP significantly strengthened the hydrogen bond binding strength between gluten and glutenin but weakened the hydrogen bond stability of gliadin. This phenomenon may be related to the change in the proportion of β-sheets conformation of the proteins. Similarly, in the study by Li [35], guar gum enhanced the hydrogen bond interaction between gluten, promoting a more uniform and dense three-dimensional network structure, which resulted in a continuously distributed dough network. It is noteworthy that, when the AAP addition concentration was too high, the hydrogen bond binding strength of gluten decreased.

Figure 3C indicates that AAP enhances the hydrophobic interaction in gliadin, while weakening its effect on gluten and glutenin. This shows that AAP promotes a transition of gluten and glutenin towards a more compact, ordered structure where hydrophobic residues are buried [36]. The above results indicate that the binding of AAP to gluten, gliadin, and glutenin is primarily driven by hydrophobic interactions, followed by hydrogen bonds, with the role of ionic bonds being the weakest.

### 3.4. Free Sulfhydryl Group Contents Determination

As shown in Figure 3D, the -SH content of gluten decreased with increasing AAP addition, reaching the lowest level at 8% AAP. It then slightly increased at 10% AAP but remained below the control group. A similar trend was observed in glutenin. In contrast, gliadin -SH content increased progressively with higher AAP levels.

The decline in -SH content indicates that the intermolecular cross-linking effect between AAP and the three-dimensional architecture of gluten significantly enhances the strength of the gluten matrix. AAP’s hydrophilic groups likely enhance water-holding capacity, promoting -SH to S-S conversion via water-induced oxidation [36]. This strengthening of intermolecular cross-linking significantly increases the strength of covalent connections between gluten molecular chains, ultimately forming a stable network structure with higher mechanical strength. According to Li’s research [11], the addition of AAP also reduced the -SH content of gluten. The rebound in -SH at 10% AAP may result from the excessive AAP diluting the gluten, causing the gluten to depolymerize and disrupt the formation of the disulfide bond [36].

The selective reduction in -SH content in glutenin compared to gliadin provides direct evidence for the differential effects of AAP on these protein fractions. The reduction in -SH content in glutenin at 8% AAP indicates a significant promotion of disulfide bond formation, which is crucial for the formation of a stable and elastic gluten network. This is consistent with the enhanced G′ and Tp values observed for glutenin at this concentration. In contrast, the increased -SH content in gliadin suggests that AAP hinders the formation of disulfide bonds between gliadin molecules, which is consistent with gliadin’s limited ability to form intermolecular disulfide bonds due to its monomeric structure [31]. This differential effect on disulfide bond formation is directly related to the molecular structure of AAP. AAP contains hydroxyl and carboxyl groups that can interact with protein amino groups, promoting hydrogen bonding and potentially facilitating the oxidation of -SH groups to S-S bonds [12]. However, the effectiveness of this interaction depends on the protein’s structure.

Glutenin, with its high molecular weight and ability to form intermolecular disulfide bonds, provides the elastic backbone of the gluten network. The AAP’s hydrophilic nature enhances its water-holding capacity, promoting -SH to S-S conversion via water-induced oxidation [36]. This is particularly effective for glutenin, which has a higher number of accessible cysteine residues due to its extended chain structure [37]. In contrast, gliadin, with its monomeric structure and limited capacity for disulfide bond formation, is less affected by AAP’s promotion of disulfide bond formation. The hydrophilic nature of AAP may even interfere with gliadin’s natural conformational dynamics. Gliadin’s structure is maintained primarily through non-covalent interactions, including hydrogen bonds and hydrophobic interactions [31]. AAP’s strong hydrogen bonding with gliadin’s polar amino acid residues may disrupt these natural interactions, leading to a less stable structure and reduced disulfide bond formation.

### 3.5. Surface Hydrophobicity Determination

As shown in Figure 3E, the surface hydrophobicity (H0) of glutenin was higher than that of gluten and gliadin. With increasing AAP addition, H0 of gluten and glutenin first decreased and then increased, dropping from 98.41 and 152.86 (control) to minima of 79.51 and 99.43 at 8% AAP. At 10% AAP, H0 increased but remained below control levels. In contrast, the H0 of gliadin steadily increased with the addition of AAP.

The higher H0 of glutenin likely stems from its abundance of non-polar amino acids, such as proline and leucine, which enhance hydrophobicity and form an elastic network via hydrophobic interactions and disulfide bonds [37,38]. The decrease in H0 for gluten and glutenin may result from the rearrangement of the molecular structure of gluten and glutenin caused by the addition of AAP, resulting in the protein being in an aggregated state and folding, causing the hydrophobic groups to be buried inside and the binding sites of bromophenol blue (BPB) to decrease [39]. Similar effects were observed with high-molecular-weight inulin, which lowers glutenin H0 by reducing hydrophobic exposure [40]. It is also possible that AAP is a hydrophilic colloid with viscosity, which is encapsulated on the surface of gluten and glutenin, not only enhancing the polarity of the protein-polysaccharide complex system but also shielding the hydrophobic groups on the surface of gluten and glutenin, playing a certain role in reducing H0 [41].

When the AAP addition amount is 10%, the H0 of gluten and glutenin increases, but is still less than that of the control group. At high concentrations of AAP, the structure of gluten and glutenin expands, and the hydrophobic groups buried in the protein are exposed, increasing H0. The H0 of gliadin increases with the addition of AAP. This may be due to the polar amino acids of AAP binding to the hydrophilic regions of gliadin, thereby breaking the original intramolecular hydrogen bonds of gliadin and causing the structure to expand. Although AAP introduces a large number of hydrophilic groups on the surface of gliadin, leading to an increase in the polarity of the protein-polysaccharide complex system, the detection of surface hydrophobicity is mainly related to the exposure degree of hydrophobic groups on the protein surface, and the influence of AAP-induced protein structure changes is greater than the contribution of the colloid’s hydrophilicity.

### 3.6. Effect of AAP on Secondary Structures of the Samples

**Figure 4:**
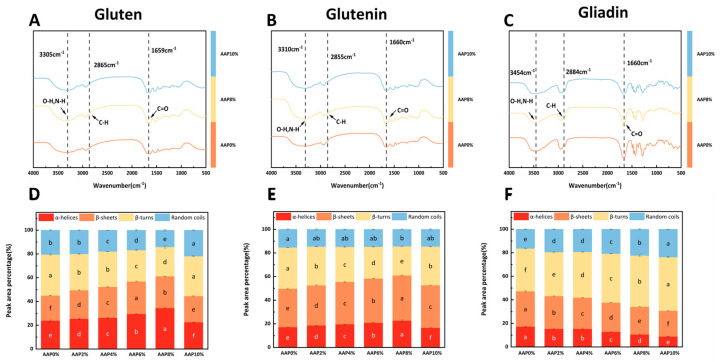
The effects of different amounts of AAP addition on the Fourier-transform infrared spectroscopy of gluten, glutenin, and gliadin, as well as the secondary structures of (**A**–**C**), representing the Fourier-transform infrared spectroscopy of gluten, glutenin, and gliadin, respectively, and (**D**–**F**), representing the secondary structures of gluten, glutenin, and gliadin, respectively). Different lowercase letters indicate significant differences, *p* < 0.05.

To present the impact of AAP on protein structure more intuitively, we analyzed the FTIR spectra of three AAP concentrations: 0%, 8%, and 10%. As shown in Figure 4A–C, no novel absorption bands were discerned in the FTIR spectra of the three proteins following AAP addition, which implies that AAP induces no significant alterations to the protein secondary structure within the detection limits of FTIR.

FTIR analysis revealed distinct concentration-dependent spectral changes upon the addition of AAP. In the 3200–3600 cm^−1^ region, the peak intensities of gluten (A) and gliadin (B) generally increased with rising AAP concentration. This trend suggests the formation of intermolecular hydrogen bonds between AAP hydroxyl groups and protein amino groups [12]. Concomitantly, the attenuation of peaks in the 2800–2900 cm^−1^ region for both proteins indicated the concealment of hydrophobic groups, which aligns with the surface hydrophobicity result. In contrast, gliadin (C) exhibited a divergent behavior: its intensity in the 3200–3600 cm^−1^ region decreased with AAP concentration; meanwhile, the increased intensity in the 2800–2900 cm ^−1^ region implied AAP-induced exposure of hydrophobic groups.

The addition of AAP significantly modulated the secondary structure of gluten. As shown in Figure 4D, AAP supplementation (≤8%) notably enhanced the contents of α-helices and β-sheets while reducing β-turns and random coils in gluten, with the ordered structure. Specifically, for glutenin, α-helices and β-sheets increased with AAP addition, peaking at 8% supplementation (31.93% and 17.72% higher than the control, respectively; Table 2). β-turns decreased (except at 10% AAP), while random coils showed no significant change, suggesting a potential conversion of β-turns to β-sheets. In contrast, AAP exerted a negative effect on gliadin’s secondary structure: Figure 4F shows that β-sheets content decreased with a concurrent increase in β-turns, whereas α-helices and random coils decreased. This indicates AAP induces the conversion of β-sheets to β-turns in gliadin, thereby reducing protein stability and promoting a more disordered structure.

When the additional amount of AAP does not exceed 8%, the contents of α-helices and β-sheets in gluten and glutenin increase significantly. Improving dough elasticity, strength, and resistance to deformation during processing. This is particularly important for applications where dough needs to maintain its structure during baking or extrusion, as well as for the final product’s texture and quality. Therefore, AAP is beneficial for enhancing the stability and elasticity of the gluten structure, which is consistent with the rheological properties of gluten. Li et al. [11] reported that the addition of AAP significantly increased the β-sheet content of gluten by 4.41%. The decrease in β-turns and increase in β-sheets could be influenced by AAP’s hydrophilic groups (hydroxyl and carboxyl), which possess strong water absorption properties. Changes in hydration can disrupt existing hydrogen bond networks that stabilize β-turns, promoting intermolecular interactions such as hydrophobic interactions and electrostatic attractions. This would favor the formation of low-molecular-weight subunit aggregates, thereby increasing the relative content of β-sheets and decreasing β-turns [6].

Different from gluten and glutenin, the content of β-sheets in gliadin decreases with the addition of AAP, while the content of β-turns increases. This indicates that AAP promotes the conversion of β-sheets to β-turns, reduces gliadin stability, and the structure of gliadin begins to transform towards a disordered state. Guo et al. [40] found that high molecular weight inulin reduced α-helices and β-sheets in gliadin, a different outcome than observed here. The reconstruction of the gluten matrix is influenced by the conformational synergy of subunit components (glutenin/gliadin).

**Figure 5:**
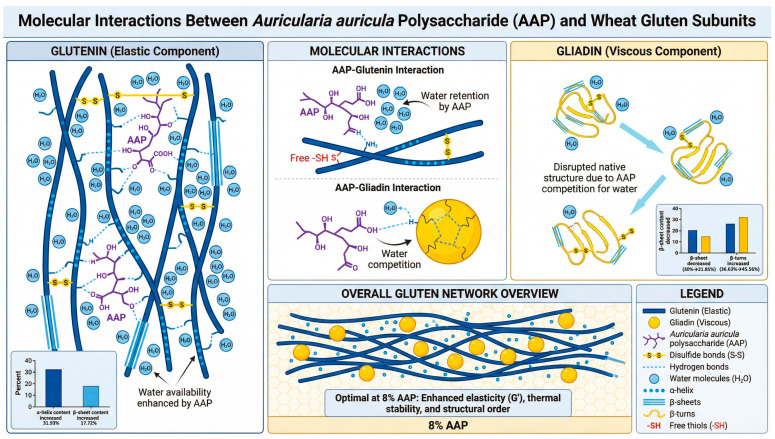
The mechanism diagram of AAP’s influence on wheat gluten and its subunit components’ secondary structures.

Figure 5 indicates that the addition of AAP alters the molecular microenvironment of glutenin and gliadin, increasing the number of disulfide bonds and enhancing their function. Ultimately, this results in a more compact structure of gluten, resulting in a uniform and dense network structure. AAP promotes an increase in the α-helices and β-sheets content of glutenin but reduces the orderliness of the secondary structure of gliadin. This process, in coordination with the synergistic effect of hydrophobic interactions and ionic bonds, prompts glutenin to shift towards a more stable state, significantly enhancing the orderliness of the glutenin’s secondary structure, while the disorderliness of gliadin increases. The establishment of the conformational stability of gluten mainly stems from the enhanced orderliness of the secondary structure domains of glutenin.

### 3.7. Determination of Intrinsic Fluorescence Spectra

**Figure 6:**
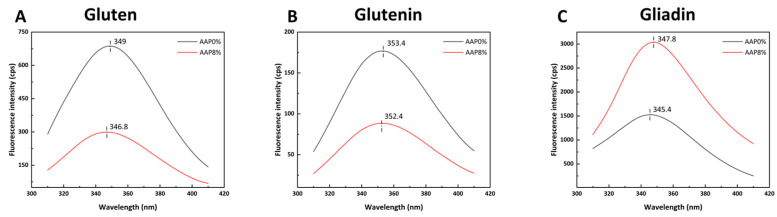
The effects of different amounts of AAP on the fluorescence spectra of gluten, glutenin, and gliadin; (**A**–**C**) represent the fluorescence spectra of gluten, glutenin, and gliadin, respectively.

Based on the above experimental results, we found that when the AAP addition amount is 8%, the viscoelasticity and thermal stability of gluten are significantly improved compared to the control sample. Therefore, we selected an 8% AAP addition amount and compared it with the control group. Figure 6A,B shows that the addition of AAP decreases the fluorescence intensity of gluten and glutenin, accompanied by a blue shift in the maximum emission wavelength (λ_max). In contrast, Figure 6C shows that AAP increases the fluorescence intensity of gliadin, accompanied by a red shift in λmax.

The decrease in fluorescence intensity and blue shift observed for gluten and glutenin may be attributed to the formation of stable complex structures through AAP binding to gluten and glutenin, which significantly reduces the content of fluorescently emitting tryptophan residues in the system, thereby causing fluorescence quenching [42]. It is also possible that AAP induces protein folding, causing amino acid residues to become buried within the protein, which results in a decrease in fluorescence intensity. In addition, AAP introduces fluorescent quenching groups [41]. The blue shift phenomenon indicates that the exposure of tryptophan residues decreases, the non-polar nature of the microenvironment of gluten increases, and more hydrophobic groups are exposed, promoting the aggregation of gluten under hydrophobic interactions. This result is consistent with the conclusions regarding the secondary structure of gluten, the content of -SH groups, and surface hydrophobicity. Similar effects were reported by Guo [40], who observed a decrease in glutenin fluorescence with increasing medium-molecular-weight inulin concentration.

For gliadin, the increased intensity and red shift suggest that AAP binds to gliadin through hydrophobic interactions or hydrogen bonds, causing conformational changes, structural disintegration in a loose state, and the exposure of tryptophan residues within the internal hydrophobic core. The red shift phenomenon indicates that the protein aggregates disintegrate, and the internal hydrophobic interaction weakens. This may be because AAP is hydrophilic, altering the solvent accessibility of the local region of the gliadin and exposing more tryptophan residues to water molecules, resulting in a red shift of λmax.

### 3.8. Microscopic Analysis of the Protein Network

**Figure 7:**
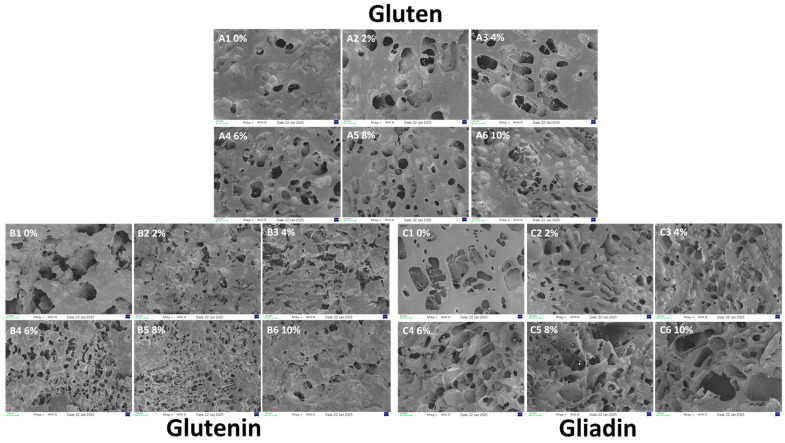
The influence of different amounts of AAP addition on the microstructure of gluten, glutenin, and gliadin. (**A1**–**A6**) represent the addition of 0–10% AAP in the gluten. (**B1**–**B6**) represent the addition of 0–10% AAP in the glutenin. (**C1**–**C6**) represent the addition of 0–10% AAP in the gliadin.

Figure 7 illustrates the impact of varying AAP concentrations on the microstructure of gluten, glutenin, and gliadin. For gluten (Figure 7(A1–A6)), as concentration increases, the network becomes more uniform and dense with smaller pores, peaking at 8% (Figure 7(A5)). At 10% (Figure 7(A6)), pores enlarge and decrease in number, disrupting the matrix. For glutenin (Figure 7(B1–B6)), increasing AAP transforms it into a more even network with smaller pores, especially at 8%. At higher concentrations, the structure opens and aggregates into blocks. For gliadin, the addition of AAP progressively destroys pores, making the structure more open, rough, and fragmented into sheets.

The denser gluten network at lower AAP concentrations likely results from hydrogen bonding between AAP’s hydroxyl groups and gluten molecules, promoting cross-linking during matrix formation. This is consistent with the research of Li [11], which shows that AAP induces the reorganization of protein network space and enhances the overall structure of the dough during the formation and water migration process. At 10%, AAP may compete for water, preventing complete gluten hydration and gluten matrix integrity.

Similar to gluten, AAP fills loose pores and forms hydrogen bonds or non-covalent interactions, causing the structure of glutenin to gradually transform from the original sheet-like structure into a network structure as the concentration of AAP increases. Due to the loose and open structure of glutenin, AAP fills the pores of glutenin, combining with it through hydrogen bonds and non-covalent interactions, forming a finer and more evenly distributed pore-like network structure. However, Excess AAP induced the structure of glutenin to become open and aggregate in a block-like form, possibly related to the gelation property of AAP. High concentrations of AAP increase its gel strength. As individual molecules overlap, the intermolecular cross-linking density increases, forming a more compact intermolecular connection network that significantly enhances the mechanical strength of the AAP network. AAP adheres to the surface of glutenin, hindering the formation of intramolecular disulfide bonds of glutenin, weakening the structure of glutenin [23,43]. This finding aligns with Guo [40], who discovered that low-molecular-weight inulin enhances glutenin structure at low doses but induces rupture and instability at high doses, demonstrating concentration-dependent effects.

For gliadin, AAP disrupts pores and induces roughness. This may be due to the presence of AAP hindering the formation of intramolecular disulfide bonds of gliadin. Similar to the findings of Guo [7], they found that konjac glucomannan disrupts the network structure of gliadin, rendering its structure rough and severely damaged. Therefore, in summary, the influence of AAP on the structure of gluten is mainly significantly related to its conformational changes to glutenin, and its structural modification effect on gliadin is relatively limited.

## 4. Conclusions

This study investigated the effects of *Auricularia auricula* polysaccharide (AAP) on the rheological, thermal, and structural properties of wheat gluten and its subunits (glutenin and gliadin) using dynamic rheology, DSC, FT-IR, SEM, and chemical interaction analyses. The results demonstrated that AAP selectively regulates glutenin to enhance gluten’s overall properties, achieving optimal effects at 8% concentration: (1) AAP increased storage (G′) and loss (G″) moduli of gluten and glutenin, promoting a denser elastic network; (2) AAP elevated denaturation temperature (Tp) and enthalpy (ΔH) of gluten and glutenin, improving thermal stability, while has a negative effects of gliadin; (3) AAP enhanced glutenin’s α-helix and β-sheet contents, strengthening ordered secondary structures and disulfide bonds, but disrupted gliadin’s structure. These findings elucidate the molecular mechanisms by which AAP modulates gluten subunits, supporting its application as a gluten modifier to improve flour product quality.

## Figures and Tables

**Table 1:** The effects of different AAP addition amounts on the changes in the thermodynamic properties of gluten, glutenin, and gliadin. Different letters in the same column indicate significant differences, *p* < 0.05.

Sample	AAPcontent (%)	Tp (°C)	ΔH (J/g)
	0	52.43 ± 0.13 f	55.47 ± 0.07 f
	2	53.21 ± 0.19 e	58.4 ± 0.06 e
Gluten	4	53.54 ± 0.06 d	81.55 ± 0.26 d
	6	55.43 ± 0.05 c	87.4 ± 0.1 b
	8	57.24 ± 0.09 a	102.01 ± 0.1 a
	10	56.31 ± 0.06 b	85.64 ± 0.09 c
	0	53.11 ± 0.09 f	69 ± 0.08 f
	2	53.3 ± 0.09 e	70.36 ± 0.04 e
Glutenin	4	53.66 ± 0.06 d	86.79 ± 0.05 d
	6	54.54 ± 0.05 c	107.6 ± 0.04 a
	8	57.37 ± 0.1 a	103.3 ± 0.11 c
	10	56.8 ± 0.07 b	103.17 ± 0.06 b
	0	54.09 ± 0.08 a	95.32 ± 0.07 a
	2	52.82 ± 0.1 b	82.42 ± 0.2 b
Gliadin	4	51.66 ± 0.13 c	77.51 ± 0.09 c
	6	51.24 ± 0.1 d	72.18 ± 0.21 d
	8	50.53 ± 0.06 e	64.81 ± 0.12 e
	10	49.78 ± 0.14 f	52.31 ± 0.17 f

**Table 2:** The influence of different amounts of AAP addition on the percentage of secondary structure of gluten, glutenin, and gliadin. Different letters in the same column indicate significant differences, *p* < 0.05.

Sample	AAPcontent (%)	α-Helices	β-Sheets	β-Turns	Randomcoils
	0	23.93 ± 0.51 e	21.16 ± 0.15 f	34.43 ± 0.51 a	20.48 ± 0.45 b
	2	25.53 ± 0.41 d	23.96 ± 0.37 d	30.51 ± 0.52 b	20 ± 0.36 b
Gluten	4	26.33 ± 0.49 c	26 ± 0.4 c	29.77 ± 0.2 b	17.9 ± 0.1 c
	6	29.56 ± 0.2 b	27.31 ± 0.3 a	26.36 ± 0.6 c	16.77 ± 0.55 d
	8	34.5 ± 0.72 a	26.74 ± 0.15 b	24.6 ± 0.96 d	14.16 ± 0.66 e
	10	22.6 ± 0.1 f	22 ± 0.35 e	33.53 ± 0.46 a	21.87 ± 0.2 a
	0	17.23 ± 0.25 e	32.56 ± 0.4 e	34.86 ± 0.15 a	15.35 ± 0.3 a
	2	18.67 ± 0.2 d	33.93 ± 0.41 d	32.8 ± 0.26 b	14.6 ± 0.62 ab
Glutenin	4	19.66 ± 0.15 c	35.89 ± 0.52 c	29.62 ± 0.5 c	14.83 ± 0.2 ab
	6	20.94 ± 0.1 b	37.43 ± 0.15 b	26.87 ± 0.26 d	14.76 ± 0.23 ab
	8	22.73 ± 0.65 a	38.33 ± 0.25 a	24.56 ± 0.3 e	14.38 ± 0.37 b
	10	16.7 ± 0.2 f	36 ± 0.2 c	32.77 ± 0.3 b	14.53 ± 0.5 ab
	0	17.33 ± 0.3 a	30 ± 0.75 a	36.36 ± 0.36 f	16.3 ± 0.2 e
	2	15.44 ± 0.45 b	27.8 ± 0.1 b	37.28 ± 0.14 e	19.48 ± 0.25 d
Gliadin	4	15.39 ± 0.61 b	26.54 ± 0.16 c	38.78 ± 0.33 d	19.29 ± 0.15 d
	6	12.89 ± 0.2 c	24.76 ± 0.06 d	41.62 ± 0.13 c	20.73 ± 0.22 c
	8	10.66 ± 0.25 d	23.51 ± 0.2 e	43.43 ± 0.83 b	22.4 ± 0.45 b
	10	8.88 ± 0.11 e	21.85 ± 0.42 f	45.56 ± 0.4 a	23.71 ± 0.09 a

## Data Availability

The original contributions presented in this study are included in the article. Further inquiries can be directed to the corresponding author.
